# Association between triglyceride-glucose index and *Helicobacter pylori* infection: a cross-sectional study

**DOI:** 10.3389/fendo.2025.1443705

**Published:** 2025-04-22

**Authors:** Dan Long, Chenhan Mao, Yin Xu, Ying Zhu

**Affiliations:** ^1^ Department of Gastroenterology, The First Hospital of Hunan University of Chinese Medicine, Changsha, Hunan, China; ^2^ Affiliated Hospital of Integrated Traditional Chinese and Western Medicine, Nanjing University of Chinese Medicine, Nanjing, Jiangsu, China

**Keywords:** triglyceride-glucose (TyG) index, *Helicobacter pylori* (H. pylori), insulin resistance, National Health and Nutrition Examination Survey (NHANES), cross-sectional study

## Abstract

**Background:**

Mounting research suggests that insulin resistance (IR) is associated with *Helicobacter pylori* (*H. pylori*) infection. The triglyceride-glucose (TyG) index has received widespread attention due to its high sensitivity in assessing IR. This study examined the association between *H. pylori* infection and TyG index.

**Methods:**

This cross-sectional study used data from the National Health and Nutrition Examination Survey (NHANES) 1999 - 2000. Participants were categorized into quartile groups (Q1–Q4) based on their TyG index. Weighted multivariable-adjusted logistic regression and subgroup analysis were used to explore the correlation between TyG index and *H. pylori* infection. Furthermore, sensitivity analysis was conducted to assess the robustness of our findings.

**Results:**

This study included 2,918 participants, 1,101 of whom were infected with *H. pylori*. The mean TyG index for all participants was 8.56 ± 0.67. Patients who were *H. pylori* positive had higher levels of TyG index compared with *H. pylori* seronegative participants (8.74 ± 0.03 vs. 8.57 ± 0.03, *P* < 0.05). The fourth quartile of the TyG index showed the highest odds of *H. pylori* infection compared to Q1 (OR = 2.37, 95%CI: 1.52 to 3.71, *P*  <  0.001). Sensitivity analysis indicated that the association between TyG index and *H. pylori* infection remained strong even after excluding participants with cardiovascular diseases or taking lipid-lowering medications, as well as patients with diabetes or taking glucose-lowering medications.

**Conclusions:**

In this study, a stable and strong positive association was found between TyG index and *H. pylori* infection. IR may be significantly associated with *H. pylori* infection. Further studies are necessary to elucidate the underlying mechanisms and potential clinical implications of these findings.

## Introduction

*Helicobacter pylori* (*H. pylori*), a gram-negative bacterium, which infects more than half of the worldwide population and has been identified as a global public health threat (1). *H. pylori* was classified as a class I carcinogen by the World Health Organization in 1994. It has been demonstrated that *H. pylori* infection may increase the risk of gastric diseases such as acute gastritis and peptic ulcer, and possibly promote the development of gastric cancer (2–4). Notably, there is growing evidence indicating a close association between *H. pylori* infection and diseases outside the gastrointestinal tract (5–7). When colonizing gastric epithelial cells, *H. pylori* not only induces local tissue inflammation or malignant transformation, but also leads to systemic and local changes in host metabolism. There is an intricate interaction between *H. pylori* and the regulation of body metabolism (8). In particular, the association between *H. pylori* infection and diabetes mellitus (DM) has attracted widespread academic attention (9, 10). Insulin resistance (IR) probably plays a key role in their association (11). IR is a precursor to type 2 diabetes mellitus (T2DM) and has been generally recognized as a unique and reliable measure. Therefore, it is crucial to understand the correlation between IR and *H. pylori* infection.

There is increasing evidence suggesting a significant association between IR and *H. pylori* infection (12–14). The triglyceride-glucose (TyG) index is a marker used to assess IR (15, 16). The TyG index has been validated as a reliable and convenient marker for IR (16). The advantages of the TyG index have been demonstrated in a number of diseases such as cardiovascular disease (17), diabetes (18), and hearing impairment (19). Zheng et al. (20) reported a significant association between higher TyG index and higher risk of kidney stone and its recurrence. Identifying new risk factors or predictive markers for H. pylori infection could have a profound impact on its early detection, prevention, and management. Given the association between IR and *H. pylori* infection, uncovering the relationship between the TyG index and *H. pylori* infection may contribute to a better understanding of the underlying pathophysiological mechanisms connecting metabolic disorders and infectious diseases. This could open new avenues for research and potentially lead to the development of novel treatment strategies.

## Methods

### Study design and participants

This cross-sectional study utilized data from the National Health and Nutrition Examination Survey (NHANES), a publicly available database that employs a stratified, multistage probability sampling design to capture nationally representative samples of the nonhospitalized population (21). The survey component includes demographic data, diet, questionnaires and physical examinations, as well as laboratory tests supervised by trained medical staff. In addition, NHANES utilizes a variety of modern equipment to make data collection more reliable and efficient. All raw data used in this study were extracted from the official NHANES website (https://www.cdc.gov/nchs/nhanes/). The NHANES protocol was approved by the National Center for Health Statistics (NCHS) Research Ethics Review Board and written informed consent was obtained from each participant.

**Figure 1:**
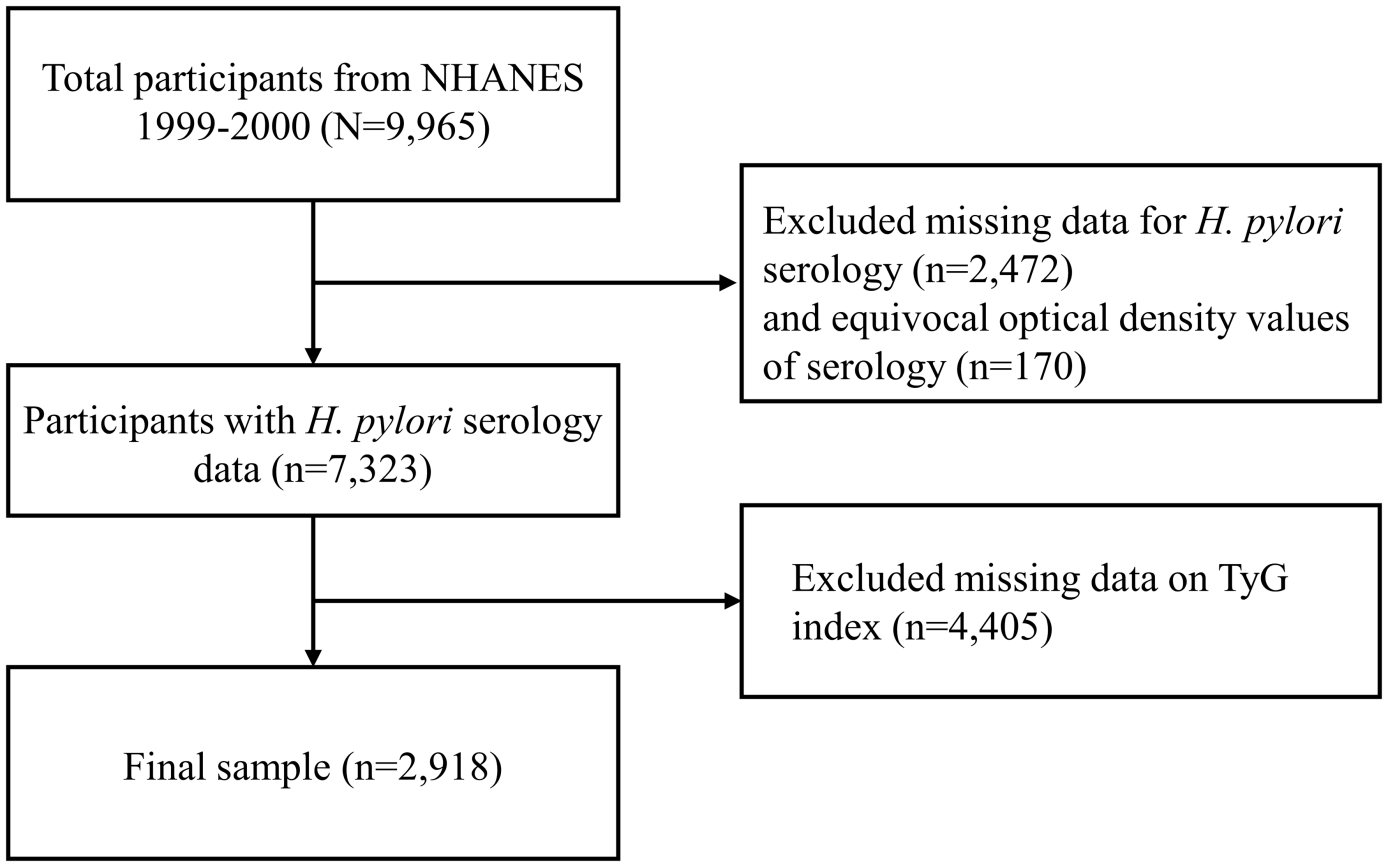
Flow chart for inclusion and exclusion of the study participants.

The NHANES 1999-2000 cycle was selected as it is the only survey period that included laboratory measurements for *H. pylori*, encompassing a total of 9,965 participants. The sample size for this study was determined by the number of eligible participants in the NHANES 1999-2000 dataset who met the inclusion criteria. A total of 2,918 participants were included in the study after excluding 2,472 individuals with missing *H. pylori* data, 170 with ambiguous *H. pylori* results, and 4,405 lacking triglyceride or glucose measurements. The inclusion and exclusion criteria are shown in **Figure 1**.

### Exposure and outcome definitions

#### Assessment of TyG index

The TyG index was calculated by TyG = ln [fasting triglyceride (mg/dL) × fasting blood glucose (mg/dL)/2] (22). The concentrations of triglyceride and fasting blood glucose (FBG) were measured by enzymatic assay using an automated biochemical analyzer. Participants were required to fast for at least 8 hours but no more than 24 hours prior to the measurement of glucose and lipid. Notably, the TyG index was considered as an exposure variable within the framework of the design of this study.

#### *H. pylori* status

*H. pylori* infection was defined as an outcome variable. *H. pylori* was evaluated by detection of immunoglobulin G (IgG) antibody using Enzyme-Linked Immunosorbent Assay (ELISA) (23). Standard ELISA cut-offs were employed to categorize participants into *H. pylori* seropositive (optical density (OD) value ≥ 1.1) or seronegative (OD value < 0.9). Equivocal values (0.9 - 1.1) were excluded from the analysis to ensure accurate statistical outcomes in this study (23).

### Assessment of covariates

The study incorporated a variety of covariates to explain potential confounders. These covariates included a range of demographic and health-related variables, including age (in years), sex, race, education level, poverty-to-income ratio (PIR), smoking status (never/former/now), alcohol use (yes/no), hypertension, diabetes, cardiovascular diseases (CVD), and body mass index (BMI). Missing covariate data were handled using multiple imputation methods. Subgroup analysis was performed by dividing age into two groups (<60 and ≥60). Race included Mexican American, non-Hispanic white, non-Hispanic black, and other race. Educational levels were categorized as below high school, high school or above high school. Participants were categorized into normal (<25 kg/m^2^), overweight (25 to <30 kg/m^2^), and obese (≥30 kg/m^2^) groups based on BMI. Hypertension was diagnosed by systolic blood pressure 140 mmHg and/or diastolic blood pressure ≥90 mmHg, or self-reported physician diagnosis of hypertension, or self-reported use of hypertension medication. Diabetes was diagnosed by glycated hemoglobin A1c (HbA1c) ≥ 6.5%, or self-reported physician diagnosis of diabetes, or self-reported use of glucose-lowering medications.

### Statistical analysis

This study incorporates complex sample design and sample weights in accordance with NHANES analytic standards. Weighted baseline characteristics of participants were compared through *H. pylori*-negative and *H. pylori*-positive patients. Continuous variables were expressed as mean ± standard error (SE), while categorical variables were reported as frequency and weighted percentage. The baseline characteristics among the different groups were compared using Chi-square test, Student’s t test, and Fisher’s exact test, as appropriate (24). Based on previous studies, all participants population was divided into four groups (Quartile 1 (Q1), Q2, Q3, Q4) based on the quartiles of TyG index (25, 26). The logistic regression analysis used Q1 as the baseline reference category. The utilization of quartile categorization assists in detecting possible non-linear association and threshold effects between TyG index and *H. pylori* infection. Weighted multivariate logistic regression models were used to assess the independent association between *H. pylori* infection and TyG index in four different models. In Model 1, covariates were not adjusted. Model 2 was adjusted for age, sex, and race. Model 3 was adjusted for age, sex, race, education, BMI, and PIR. Model 4 was further adjusted for potential confounders, including age, sex, race, education, BMI, PIR, smoking, alcohol use, hypertension, DM, and CVD. Regression analysis results were reported as odds ratio (OR) values and 95% confidence intervals (CIs). Moreover, subgroup analyses were performed to evaluate possible heterogeneity. Interactions with age, sex, race, and BMI were tested. Subgroup covariates were analyzed using fully adjusted Model 4. To assess the robustness of our findings, sensitivity analyses were conducted by excluding individuals with cardiovascular disease, those using lipid-lowering medications, patients with diabetes, or those receiving glucose-lowering therapies. In addition, the relationship between FBG and *H. pylori* seropositivity was analyzed using weighted multivariable logistic regression analysis and compared with the TyG index.

All analyses were performed using R software (R version 4.3.2). *P* < 0.05 was considered statistically significant.

## Results

### Baseline characteristics of study participants

The study ultimately included a sample size of 2,918 individuals, including 1,101 (37.7%) *H. pylori* seropositive patients (**Table 1**). The weighted mean age of all individuals was 41.05 ± 0.66 years, of which 50.8% were females and 49.2% were males. The average fasting glucose and triglycerides for all participants were 98.89 ± 0.88 (mg/dL) and 136.46 ± 3.61 (mg/dL), respectively. The weighted mean TyG index was 8.62 ± 0.02. The average TyG index values were significantly higher in the *H. pylori* positive group (8.74 ± 0.03) compared to the *H. pylori* negative group (8.57 ± 0.03) (*P* < 0.001). The TyG index quartiles were Q1 (6.94 < TyG ≤ 8.08), Q2 (8.08 < TyG ≤ 8.50), Q3 (8.50 < TyG ≤ 8.96), and Q4 (8.96 < TyG ≤ 12.48). In addition, the *H. pylori* positive group showed a higher age (*P* < 0.001), lower education level (*P* < 0.001), and lower PIR (*P* < 0.001) compared with the *H. pylori* negative group.

**Table 1:** Weighted baseline characteristics of participants with different *H. pylori* infection status.

Variables	Overall (N=2,918)	HP negative (N=1,817)	HP seropositive (N = 1,101)	*P* value
Age (years), mean (SE)	41.05 (0.66)	38.85 (0.73)	46.80 (0.88)	*P* < 0.001
Age, n (weighted %)				*P* < 0.001
< 60	2,218 (81.7)	1,485 (84.6)	733 (74.0)	
≥ 60	700 (18.3)	332 (15.4)	368 (26.0)	
Sex (%), n (weighted %)				0.35
Female	1,500 (50.8)	963 (51.4)	537 (49.1)	
Male	1,418 (49.2)	854 (48.6)	564 (50.9)	
Race, n (weighted %)				*P* < 0.001
Mexican American	954 (6.5)	453 (3.9)	501 (13.4)	
Non-Hispanic White	620 (11.2)	349 (8.1)	271 (19.4)	
Non-Hispanic Black	1,083 (69.4)	877 (78.8)	206 (45.0)	
Other Race	261 (12.8)	138 (9.2)	123 (22.2)	
PIR	2.94 (0.12)	3.13 (0.14)	2.43 (0.12)	*P* < 0.001
BMI (kg/m^2^)	27.08 (0.26)	26.88 (0.33)	27.59 (0.19)	<0.05
BMI, n (weighted %)				<0.05
Normal weight	1,296 (43.4)	890 (46.0)	406 (36.7)	
Overweight	857 (29.5)	478 (27.5)	379 (34.8)	
Obese	765 (27.1)	449 (26.5)	316 (28.5)	
Educational level, n (weighted %)				*P* < 0.001
Less than high school	822 (12.5)	445 (10.0)	377 (19.0)	
High school or equivalent	1,271 (42.9)	772 (40.6)	499 (48.8)	
College or above	825 (44.7)	600 (49.4)	225 (32.2)	
Hypertension, n (weighted %)				<0.05
No	2097 (71.7)	1,392 (74.0)	705 (65.7)	
Yes	821 (28.3)	425 (26.0)	396 (34.3)	
Diabetes, n (weighted %)				0.06
No	2,749 (95.2)	1,746 (95.9)	1,003 (93.3)	
Yes	169 (4.8)	71 (4.1)	98 (6.7)	
Cardiovascular disease, n (weighted %)				<0.05
No	2,734 (93.6)	1,730 (94.7)	1,004 (90.8)	
Yes	184 (6.4)	87 (5.3)	97 (9.2)	
Smoking status, n (weighted %)				0.002
Never	1,588 (50.2)	1,007 (51.4)	581 (46.9)	
Former	787 (27.3)	499 (28.6)	288 (23.9)	
Now	543 (22.5)	311 (20.0)	232 (29.2)	
Alcohol drinking, n (weighted %)				<0.05
No	421 (10.7)	237 (9.7)	184 (13.2)	
Yes	2,497 (89.3)	1,580 (90.3)	917 (86.8)	
Antidiabetic drugs, n (weighted %)				0.08
No	2,787 (96.8)	1765 (97.5)	1022 (95.1)	
Yes	131 (3.2)	52 (2.5)	79 (4.9)	
Lipid-lowering drugs, n (weighted %)				0.07
No	2,765 (93.6)	1,731 (94.4)	1,034 (91.7)	
Yes	153 (6.4)	86 (5.6)	67 (8.3)	
FBG(mg/dL), mean(SE)	98.89 (0.88)	96.80 (0.94)	104.38 (1.78)	0.001
TG (mg/dL), mean (SE)	136.46 (3.61)	131.62 (4.49)	149.15 (5.17)	<0.05
TyG index, mean (SE)	8.62 (0.02)	8.57 (0.03)	8.74 (0.03)	*P* < 0.001

HP, *Helicobacter pylori*; SE, standard error; BMI, body mass index; PIR, poverty income ratio; FBG, fasting blood glucose; TG, fasting triglyceride; TyG, triglyceride-glucose.

N are unweighted, mean (SE) and % are weighted.

### Higher TyG index is associated with higher likelihood of *H. pylori* infection

The results of weighted logistic regression were presented in **Table 2**.

**Table 2:** ORs and 95% CIs for *H. pylori* infection according to TyG index.

Variables	Model 1	Model 2	Model 3	Model 4
TyG quantile	OR (95% CI)	OR (95% CI)	OR (95% CI)	OR (95% CI)
Q1	Reference	Reference	Reference	Reference
Q2	1.31 (0.96, 1.78)	1.27 (0.85, 1.90)	1.31 (0.95, 1.82)	1.32 (0.95, 1.82)
Q3	1.46 (1.00, 2.14)	1.57 (0.97, 2.54)	1.72 (1.15, 2.58)	1.63 (1.08, 2.47)
Q4	2.03 (1.40, 2.94)	2.29 (1.43, 3.68)	2.51 (1.70, 3.71)	2.37 (1.52, 3.71)

OR, odds ratio; 95% C, 95% confidence interval; TyG, triglyceride-glucose.

Model 1: No covariates were adjusted.

Model 2: Adjusted for sex, age, and race.

Model 3: Adjusted for sex, age, race, education level, PIR, and BMI.

Model 4: Adjusted for sex, age, race, education level, PIR, BMI, hypertension, cardiovascular disease, diabetes mellitus, smoking, and drinking.

The positive association between *H. pylori* infection and the TyG index in the Q4 quartile compared with the lowest quartile (Q1) persisted in Model 2 (OR = 2.29; 95%CI: 1.43 to 3.68) and Model 3 (OR = 2.51; 95%CI: 1.70 to 3.71). After adjusting for all potential confounders in Model 4, we found a progressive and significant increase in the risk of *H. pylori* seropositivity in increasing quartiles of the TyG index. Specifically, the fourth quartile of the TyG index showed the highest odds of *H. pylori* infection compared to Q1, with an OR of 2.37 (95%CI: 1.52 to 3.71, *P* < 0.001).

### Subgroup analysis

**Figure 2:**
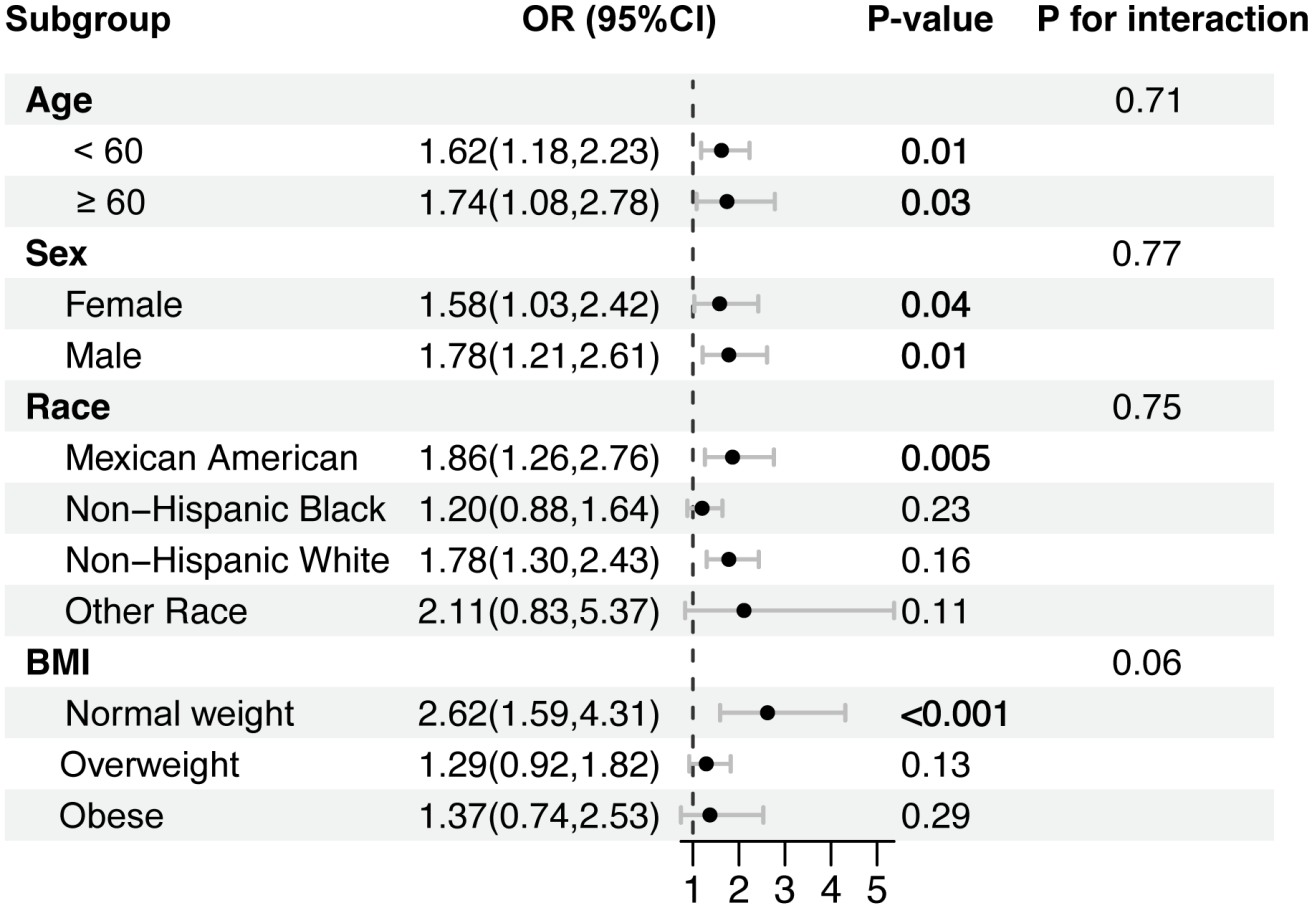
Subgroup analysis for the association between the TyG index and *H. pylori* infection.

The results of subgroup analyses are displayed in **Figure 2**. Interestingly, the positive association between TyG index and *H. pylori* infection was pronounced in participants under 60 years of age (OR = 1.58; 95% CI: 1.21 to 2.06), Mexican Americans (OR = 1.90; 95% CI: 1.32 to 2.71), non-Hispanic white (OR = 1.74; 95% CI: 1.31 to 2.32), and individuals with a BMI < 30. However, no correlation with the *P* for interaction meeting the statistical significance was detected in all subgroup analyses, indicating that the association was not dependent on age, sex, race, and BMI (*P*  >  0.05).

### Sensitivity and additional analyses

Sensitivity analysis indicated that the association between TyG index and *H. pylori* infection remained strong even after excluding participants with CVD or taking lipid-lowering medications, as well as patients with diabetes or taking glucose-lowering medications (**Supplementary Table 1**). To assess whether TyG index is more closely related to *H. pylori* infection than FBG, the relationship between FBG and *H. pylori* infection was further analyzed. Compared to the reference group in the lowest quartile, participants in the highest quartile of FBG showed an OR of 1.81 (95% CI: 1.30 to 2.51) in Model 4 (**Supplementary Table 2**), which was lower than that observed in the highest quartile of TyG index (OR = 2.37; 95%CI: 1.52 to 3.71).

## Discussion

Previous studies have reported the relevance of *H. pylori* infection to several other pathologic factors (23, 27–29). Interestingly, Xiong et al. found that higher dietary inflammatory index (DII) was associated with an increased risk of *H. pylori* infection and was related to a higher risk of all-cause mortality only in *H. pylori* infected individuals (23). The DII is a scoring system that evaluates the potential inflammatory levels of dietary components. Inflammation has been reported to be positively associated with IR (30). In addition, gastric *H. pylori* colonization has been reported to be correlated with impaired glucose tolerance (27). A Japanese cross-sectional study showed that *H. pylori* infection significantly and independently promoted IR in a large asymptomatic population (11). The TyG index, on the other hand, is a new indicator that has been used to assess IR status in recent years. The main finding of this study was that *H. pylori* infection was positively associated with TyG index. The mean value of TyG index for all participants in this study was 8.62 ± 0.02. The mean TyG index values for H. pylori seropositivity and seronegativity were 8.74 ± 0.03 and 8.57 ± 0.03, respectively. Subgroup analyses and interaction tests showed that the association was not dependent on age, sex, race, and BMI. A clinical study showed that *H. pylori* seropositivity was associated with higher homeostatic model assessment for IR (HOMA-IR) values in patients with DM (31). Besides, a NHANES-based study reported a significant association between *H. pylori* seropositivity and metabolic score for IR (METS-IR) (32). These evidences further support the correlation between *H. pylori* infection and IR. Our findings are consistent with those of previous studies but provide additional insight into the extent of the association between *H. pylori* infection and TyG index.

The potential mechanisms explaining the relationship between TyG index and *H. pylori* infection remain to be further explored, and there may be several possible explanations as follows. Firstly, *H. pylori* seropositive patients suffer from poor IR. The study revealed a correlation between *H. pylori* infection and IR in pediatric populations (33). In addition, a meta-analysis involving 206,911 individuals also demonstrated that a higher risk of *H. pylori* infection was associated with IR (13). A randomized, double-blind, placebo-controlled trial demonstrated that the eradication of *H. pylori* improved glucose homeostasis in patients with T2DM by decreasing pro-inflammatory factors and fasting insulin levels (34). Mechanistically, the higher expression of suppressor of cytokine signaling 3 (SOCS3) is thought to exacerbate IR (35, 36). *In vivo* and *in vitro* experiments have shown that *H. pylori* infection could up-regulate SOCS3 expression by down-regulating miR-203 (12). Knockdown of SOCS3 attenuates *H. pylori*-induced impairment of insulin signaling (12). It has been shown that *H. pylori* infection affects the production of metabolic hormones involved in energy homeostasis (37), which may be another potential mechanism for *H. pylori*-related IR. Interestingly, it was revealed that diet-induced IR exacerbated by *H. pylori* may be associated with gut dysbacteriosis (14). Another report emphasized that there is a continuous crosstalk between *H. pylori* and the gut microbiota, which is involved in intestinal inflammation (37). In addition, studies suggest that the role of *H. pylori* in impaired glucose tolerance may be enhanced by higher BMI levels (14, 27). Notably, *H. pylori* infection has also been reported to be associated with higher HbA1c level and the development of T2DM (27, 38). Although increasing studies have reported a positive association between *H. pylori* and IR (11, 13), large-scale prospective studies are still needed to validate their association in the future.

Secondly, reverse causality may also explain the relationship between higher levels of TyG and higher odds of *H. pylori* infection. Notably, a higher TyG index not only implies IR, but also symbolizes adverse health conditions associated with diabetes, cardiovascular disease, obesity, hypertension, metabolic syndrome, and disorders of lipid metabolism (39–41). A hospital-based case study tested fecal antigen on 148 participants and showed that patients with DM were more likely to be infected with *H. pylori* in comparison with non-diabetic individuals (42). Diabetes-induced IR and chronic inflammation impair cellular and humoral immunity in patients, thereby enhancing individuals’ susceptibility to infection (43). Indeed, diabetic patients are also associated with impaired gastric secretions, gastrointestinal motility dysfunction, and aggravated gastric mucosal atrophy (44, 45). The impaired mucosal immunity and damage to the gastric epithelium provide an environment for *H. pylori* to colonize the gastric epithelium, thus enhancing susceptibility to infection (46). Although the association between T2DM and higher rates of *H. pylori* infection is controversial (47–49), once infected with *H. pylori*, patients with T2DM may be at higher risk for gastric cancer (50). Therefore, early detection and prompt treatment of *H. pylori* infection should be a priority to reduce the risk of gastric cancer in patients with T2DM. Furthermore, a recent meta-analysis reported that *H. pylori* infection increases the risk of coronary heart disease (CHD) (51). Chronic *H. pylori* infection triggers immune responses and activates inflammatory cytokines (52). Prolonged exposure to these inflammatory cytokines induces a chronic inflammatory cascade and changes in lipid metabolism. Thus, these changes may contribute to the development of atherosclerosis. A large cohort and long-term follow-up study involving more than 1,100 subjects suggested that the eradication of *H. pylori* prevents the development of CHD (53).

## Advantages and limitations

This study presents several advantages. Firstly, this study is based on the NHANES database, which is a reliable source. Secondly, confounders were adjusted to ensure that the results of this study were convincing. Thirdly, the potential effects of hypoglycemic and lipid-lowering drugs on TyG index were fully considered in this study. Sensitivity analysis was used to assess the robustness of our findings. Moreover, we assessed whether the relationship between TyG index and *H. pylori* seroprevalence was closer than fasting glucose.

However, some limitations of this study should not be overlooked. To begin with, this paper is based on the U.S. NHANES database, which is not fully representative of populations around the world. Then, due to the cross-sectional study design, we could not identify a causal relationship between TyG index and *H. pylori* infection. Therefore, our findings must be interpreted with caution and viewed as primary evidence worthy of further study. Furthermore, the use of *H. pylori* IgG serology as a marker of infection does not distinguish between current and past infections. This may have influenced our results, as the persistence of IgG antibodies could lead to an overestimation of active infection rates. Future studies using more specific methods, such as the urea breath test, could provide more accurate assessments of current infection status. Last but not least, This study utilized laboratory data collected during NHANES 1999-2000, which may raise concerns about the accuracy of techniques used at that time. However, NHANES adheres to rigorous quality control and standardization protocols, and the methods employed were state-of-the-art and well-validated. While the age of the data is a limitation, our findings are consistent with more recent studies (54, 55), supporting the robustness of our results.

## Conclusions

Our findings demonstrate a significant positive association between TyG index levels and risk of *H. pylori* infection, suggesting its potential utility as a predictive biomarker. Nevertheless, additional large-scale studies are essential accurately determine the precise causal relationship underlying this association.

## Data Availability

Publicly available datasets were analyzed in this study. These data can be found here: https://www.cdc.gov/nchs/nhanes/.
