# Supplementary material for: Association between triglyceride-glucose index and Helicobacter pylori infection: a cross-sectional study
Source: Front Endocrinol (Lausanne). 2025 Apr 22;16:1443705. doi: 10.3389/fendo.2025.1443705 (PMC12052528; doi:10.3389/fendo.2025.1443705)
Supplement: Supplementary file 1 [file Table1.docx]

**Supplementary Table 1** Weighted multivariable logistic regression analyses of TyG index and *H. pylori* infection.

| Variables | Model 1 | Model 2 | Model 3 | Model 4 |
| --- | --- | --- | --- | --- |
| TyG index | OR (95% CI) | OR (95% CI) | OR (95% CI) | OR (95% CI) |
| Q1 | Reference | Reference | Reference | Reference |
| Q2 | 1.48 (0.89, 2.46) | 1.45 (0.76, 2.75) | 1.52 (0.93, 2.50) | 1.53 (0.95, 2.47) |
| Q3 | 1.43 (0.99, 2.05) | 1.47 (0.92, 2.35) | 1.63 (1.11, 2.38) | 1.56 (1.06, 2.30) |
| Q4 | 2.40 (1.61, 3.57) | 2.95 (1.70, 5.13) | 3.35 (2.15, 5.21) | 3.14 (1.96, 5.02) |

OR: odds ratio; 95% Cl: 95% confidence interval; TyG: triglyceride-glucose.

Model 1: No covariates were adjusted.

Model 2: Adjusted for sex, age, and race.

Model 3: Adjusted for sex, age, race, education level, PIR, and BMI.

Model 4: Adjusted for sex, age, race, education level, PIR, BMI, hypertension, smoking, and drinking.

**Supplementary Table 2** Weighted multivariable logistic regression analyses of FBG and *H. pylori* infection.

| Variables | Model 1 | Model 2 | Model 3 | Model 4 |
| --- | --- | --- | --- | --- |
| FBG quantile | OR (95% CI) | OR (95% CI) | OR (95% CI) | OR (95% CI) |
| Q1 | Reference | Reference | Reference | Reference |
| Q2 | 1.83 (1.12, 2.98) | 1.91 (1.13, 3.22) | 1.85 (1.15, 2.98) | 1.86 (1.14, 3.03) |
| Q3 | 1.74 (1.17, 2.59) | 1.76 (1.17, 2.66) | 1.74(1.20, 2.52) | 1.73 (1.21, 2.46) |
| Q4 | 2.29 (1.60, 3.27) | 1.96 (1.39, 2.76) | 1.90 (1.35, 2.67) | 1.81 (1.30, 2.51) |

FBG, Fasting blood glucose; OR: odds ratio; 95% Cl: 95% confidence interval.

Model 1: No covariates were adjusted.

Model 2: Adjusted for sex, age, and race.

Model 3: Adjusted for sex, age, race, education level, PIR, and BMI.

Model 4: Adjusted for sex, age, race, education level, PIR, BMI, hypertension, cardiovascular disease, diabetes mellitus, smoking, and drinking.
